# A complicated Chiari type 1 malformation and holocord syrinx as a likely cause for heel pain

**DOI:** 10.1007/s00381-024-06299-7

**Published:** 2024-02-01

**Authors:** Robert Henderson, Rahul Lakshmanan, Aden McLaughlin, Omar Bangash, Snigdha Saha, Richard Carey-Smith

**Affiliations:** 1 Department of Radiology, Perth Children’s Hospital, Perth, WA Australia; 2 University of Western Australia, Crawley, Perth, WA Australia; 3 Department of Radiology, Royal Orthopaedic Hospital NHS Foundation Trust, Birmingham, UK; 4 Centre for Neuromuscular and Neurological Disorders (Perron Institute), University of Western Australia, Nedlands, WA Australia; 5 Department of Neurosurgery, Perth Children’s Hospital, Perth, WA Australia; 6 Department of Orthopaedics, Perth Children’s Hospital, Perth, WA Australia; 7 Orthopaedic and Sports Medicine Centre, West Perth, WA Australia

**Keywords:** Child, Holocord syrinx, Syringomyelia, Neuropathic osteoarthropathy, Cystic cerebellar degeneration

## Abstract

**Background:**

Chiari malformations are a rare group of rhomboencephalic abnormalities involving the brain, craniocervical junction and spine. They may manifest in a variety of clinical presentations which relate to the variable involvement of the cerebellum, brainstem, lower cranial nerves, spinal cord and altered CSF flow dynamics.

**Method:**

We report an unusual case of incidental diagnosis of a type I Chiari malformation with secondary cystic cerebellar tonsillar encephalomalacia and holocord syrinx following investigation of a 5YO girl presenting with heel swelling related to progressive neuropathic osteoarthropathy of the posterior calcaneal body and apophysis.

**Result:**

The child was treated with decompressive suboccipital craniectomy and C1 laminectomy and tonsillar resection. Cerebellar tonsillar gliosis and cystic degeneration were confirmed on histopathology. Referral for ongoing engagement with occupational and physical therapy.

**Conclusion:**

Most type I Chiari malformations in the paediatric population are incidental and asymptomatic. Neurological symptoms are typically mild and relate to altered CSF flow dynamics; however, we present a complex case of type I Chiari malformation with an unusual constellation of associated complications.

## Introduction

Chiari malformations are a rare group of rhomboencephalic abnormalities involving the brain, craniovertebral junction and the spine. Although classified into four distinct subtypes [1–4], Chiari malformations may present with diverse clinical manifestations relating to variable involvement of the cerebellum, brainstem, lower cranial nerves, spinal cord and altered CSF flow dynamics.

Classified into four subtypes, the types 1–3 are associated with a varying degree of caudal displacement of posterior fossa structures, with classical herniation of the cerebellar tonsils through the foramen magnum. Type 4 Chiari malformation illustrates a cerebellar hypo/aplasia with an accompanying occipital encephalocoele [1–3]. It is unlikely that these different types of Chiari malformations share a common pathoembryological origin. Given the complexity and varying severity of these abnormalities, it is not surprising that the resultant clinical manifestations are diverse.

Type 1 Chiari malformations are defined by caudal migration of the cerebellar tonsils below the foramen magnum > 5 mm [1, 2, 4–6], with a clinical incidence of < 1% and 3:1 prevalence in females. Although approximately 50% are incidental or otherwise asymptomatic, there is a propensity for symptom severity to be proportional to the degree of caudal tonsillar displacement and Syringomyelia.

## Case report

A 5-year-old girl presented to a regional hospital Emergency Department with progressive painless swelling of her right ankle and foot. Physical examination showed a swollen ankle and hindfoot without significant erythema. There was no discernible sensorimotor deficit on the initial assessment. The patient was afebrile, and on initial investigation, inflammatory markers were normal.

**Fig. 1:**
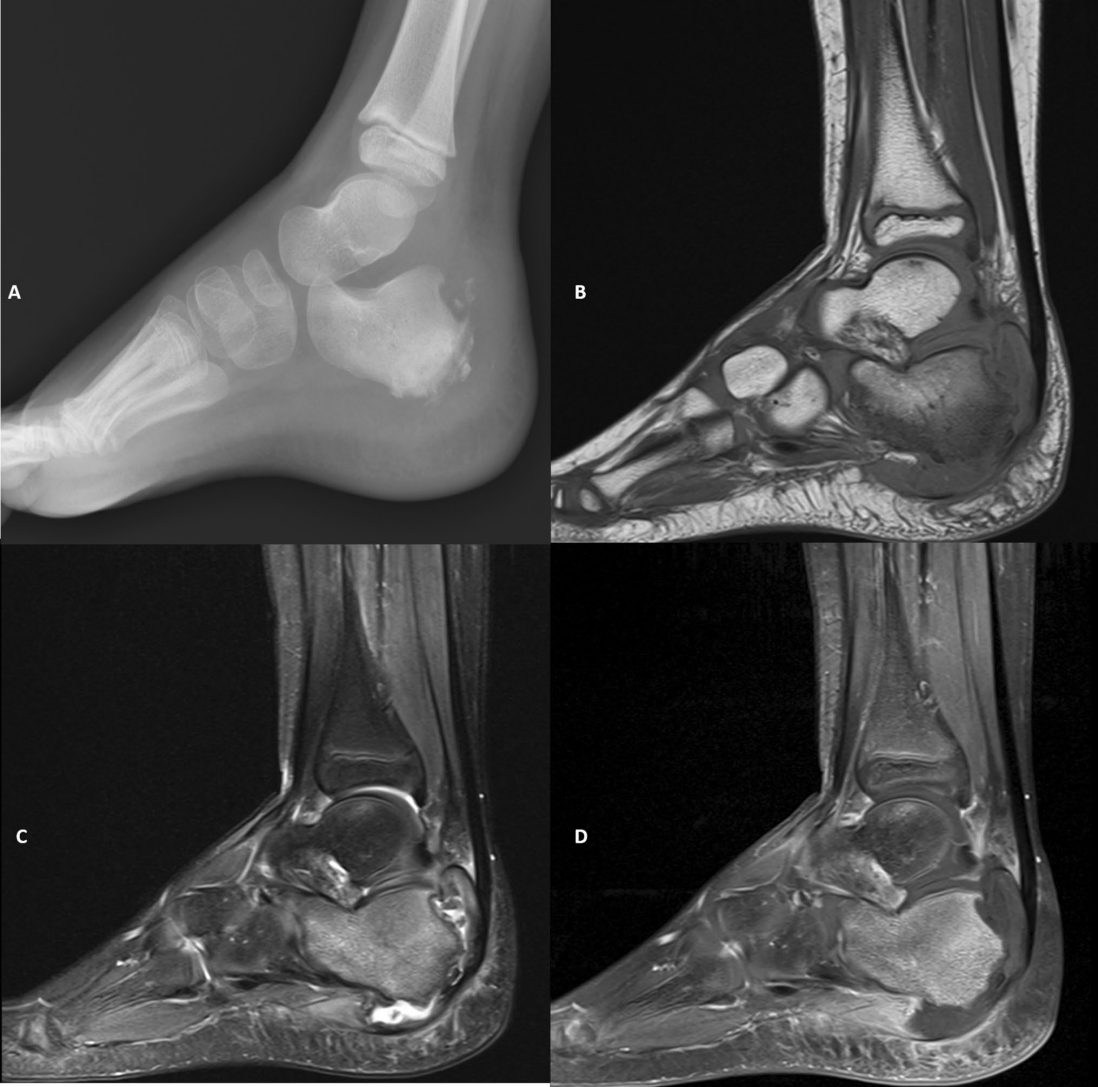
Lateral radiograph of the hindfoot at presentation (**A**) showing a sclerotic remodelling of the posterior calcaneal body with sclerosis and fragmentation of the apophysis, retrocalcaneal scalloping and moderate associated soft tissue swelling. Further evaluation with MRI with sagittal T1 and T2 weighted imaging reveals loss of normal T1 marrow signal (**B**) at the posterior calcaneus with diffuse marrow oedema (**C**) and associated contrast enhancement on post-gadolinium sequences (**D**). Soft tissue collection involving the fragmented calcaneal apophysis with a non-enhancing and low T1/T2 signal margin (**B**–**D**). Modest surrounding soft tissue oedema and enhancement at the hindfoot region

An X-ray of the ankle (Fig. 1A) was obtained which showed sclerotic change through the posterior calcaneum with destruction and fragmentation of the calcaneal apophysis. There was moderate surrounding soft tissue swelling although no ankle joint effusion.

The following day an MRI (Fig. 1B–D) revealed an irregular destructive process centred on the posterior calcaneus, calcaneal growth plate and adjacent apophysis. Fluid signal tracts with no rim enhancement extend across the growth plate and along the undersurface of the calcaneal body deep to the plantar fascia origin. There is patchy oedema and enhancement through the calcaneal bone marrow and surrounding soft tissues. No abnormal soft tissue collection or mass.

Orthopaedic incisional biopsy of the calcaneal lesion was obtained with the histopathological report identified periosteal fibrous tissue and physeal cartilage with areas of non-inflamed granulation tissue. No suppurative, chronic or granulomatous inflammation nor evidence of malignancy was present.

**Fig. 2:**
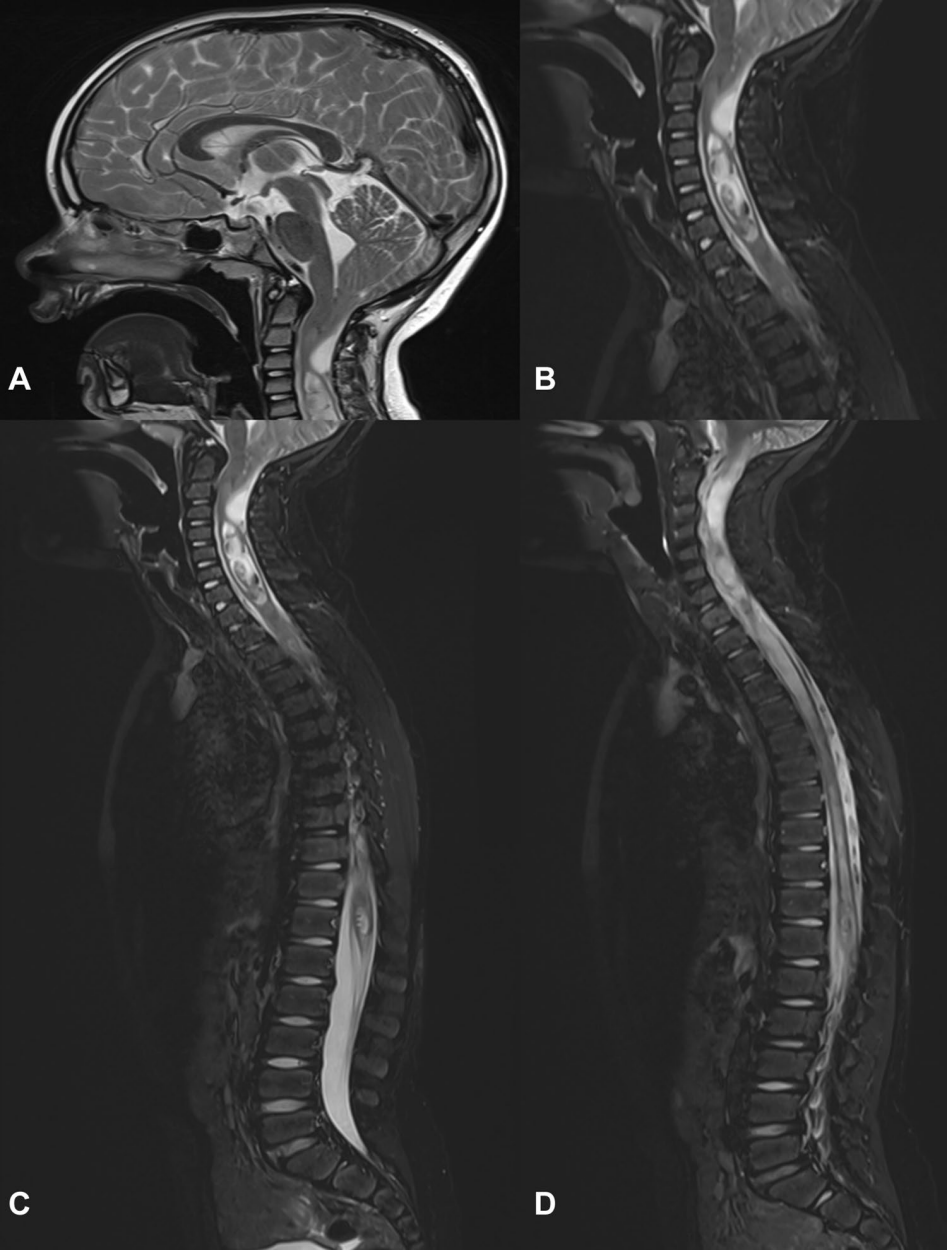
Sagittal T2 weighted images of the midline brain and spinal cord showing descent of the right cerebellar tonsil level with the C4 superior endplate (**A**) and accompanying high T2 signal changes of the tonsil (**A**, **B**) and holocord syringomyelia (**B**–**D**). There was no associated myelomeningocoele (**A**–**D**) to confirm a type 1 Chiari malformation. The odontoid process is retroflexed; however, there is no hydrocephalus (**A**)

**Fig. 3:**
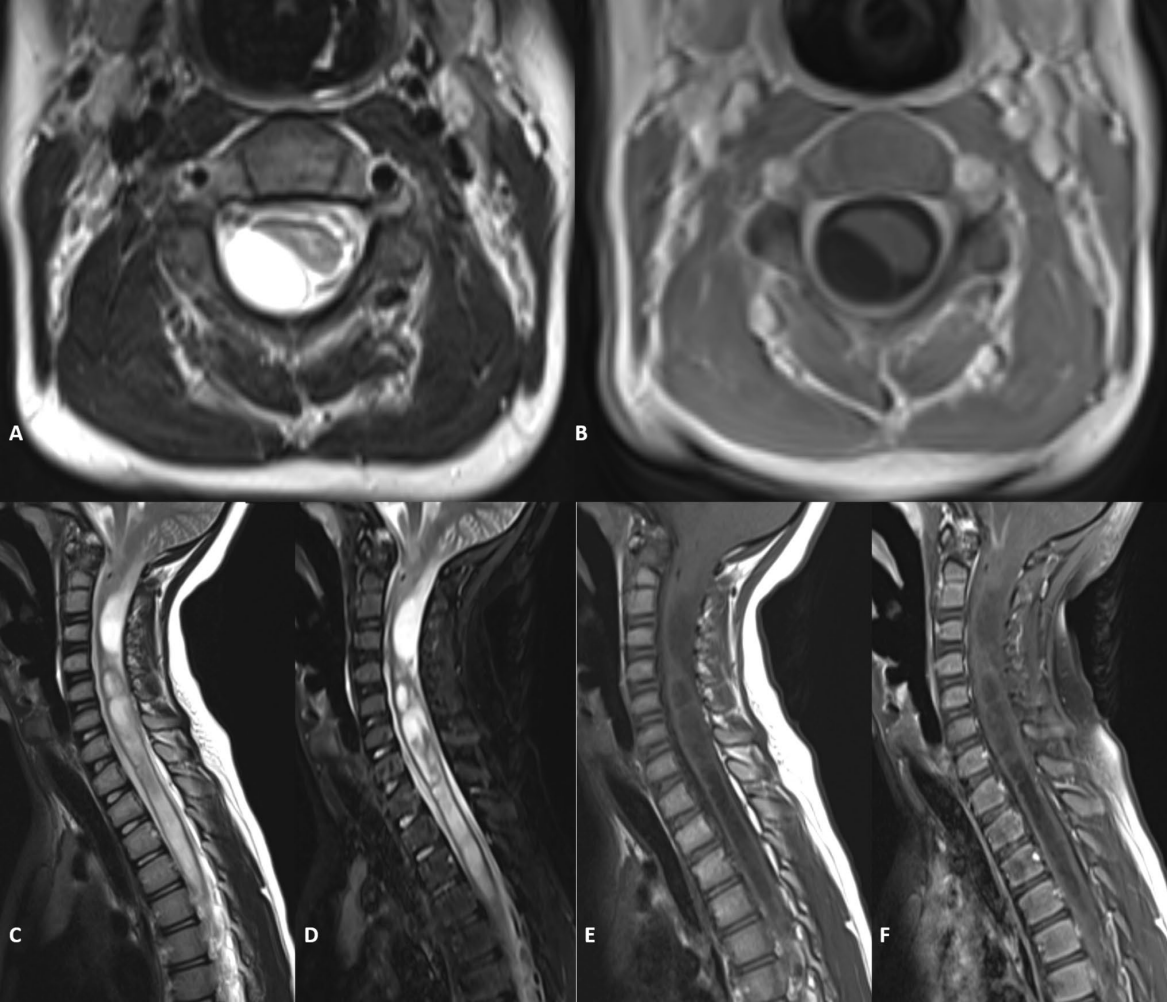
Further targeted neuroimaging showed cystic degeneration of the herniated right cerebellar tonsil compressing the dorsolateral upper cervical cord and an associated holocord syringomyelia. Axial and sagittal T2 weighted imaging of the cervical cord region showed T2 hyperintense identical to CSF with a thin and smooth low signal margin (**A**, **C**, **D**). Sagittal T1WI similarly shows low signal internal contents similar to CSF (**E**) with no demonstrable enhancement on post-gadolinium sequences (**B**, **F**)

Based on the imaging characteristics of the calcaneal lesion and an alternative diagnosis of chronic recurrent multifocal osteomyelitis provided, and hence a contemporaneous screening whole body MRI was performed. This study would unmask an unexpected Chiari 1 malformation (Fig. 2) with right tonsillar descent over 40 mm to C4 with upper cervical cord compression. This is further complicated by cystic degeneration of the right cerebellar tonsil causing additional dorsal cervical cord compression and an extensive holocord syringomyelia between C4 to the conus at T12. There was no abnormal contrast enhancement nor significant hydrocephalus (Fig. 3). No additional bone lesion was evident.

**Fig. 4:**
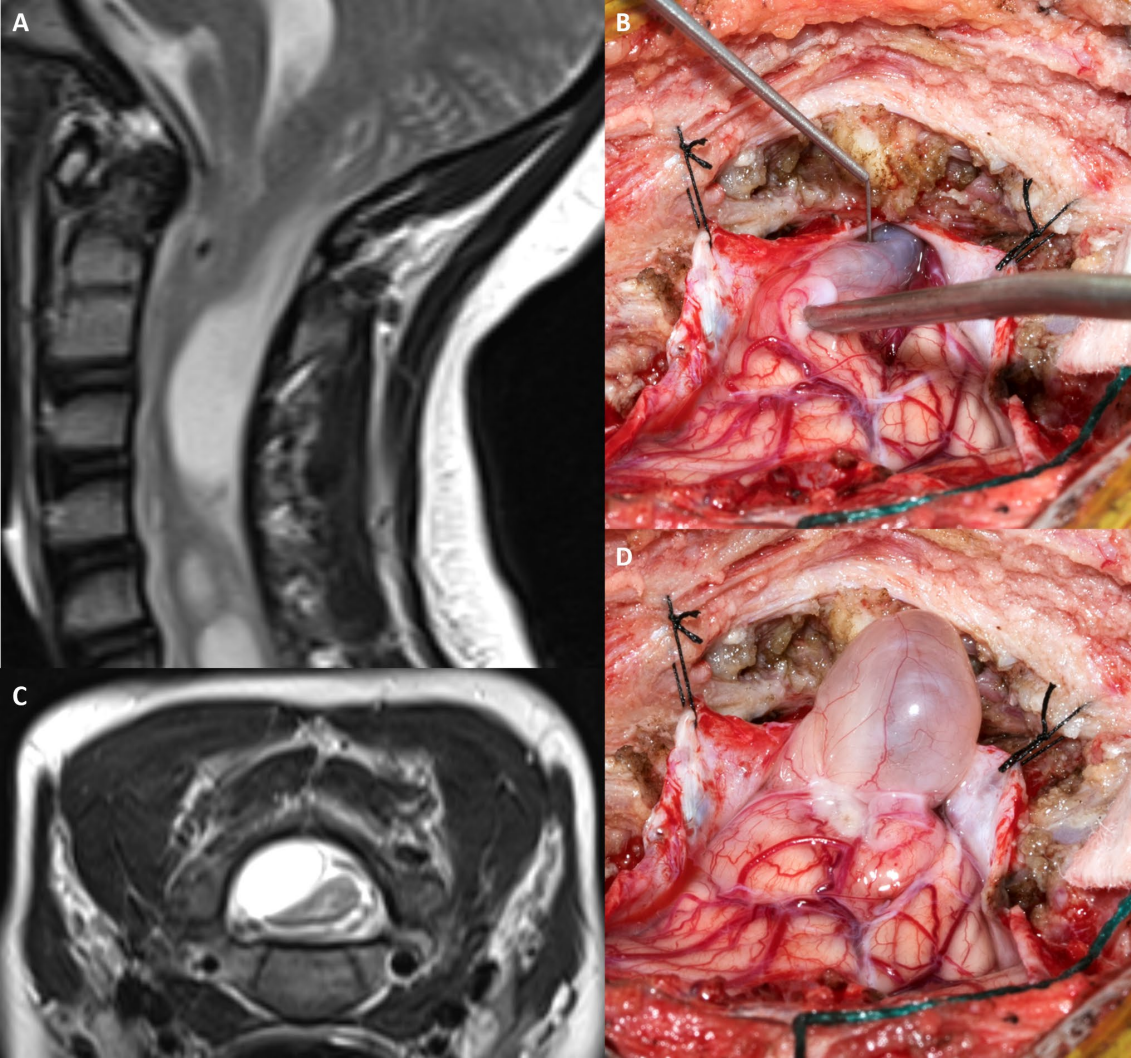
Intra-operative post-durotomy images showed cystic encephalomalacic change of the right cerebellar tonsil. This is first revealed in the upper cervical canal (**C**) corresponding to the sagittal and inverted axial T2 weight imaging (**A**, **B**) prior to its delivery into the surgical field (**D**) and subsequent resection

Following referral and review by the paediatric neurosurgical team, a suboccipital craniectomy a C1 laminectomy was performed with Chiari malformation decompression and duroplasty. The right cerebellar tonsil was reduced, with the tonsillar cyst and adjacent parenchyma resected (Fig. 4). Histopathological report identified cerebellar parenchymal gliosis with cystic change. No features of malignancy were present. Uncomplicated post-neurosurgical recovery with the patient discharged on post-operative day 5 with a complement of physical and occupational therapy follow-up.

## Discussion

Chiari 1 malformations are a rare entity, with the associated holocord syringomyelia and cerebellar tonsillar cystic degeneration which are rarer further still. Given the known association of syringomyelia and neuropathic (Charcot) osteoarthropathy, this is the likely cause for the calcaneal changes, hence rendering this a complex multisystem case.

### Cystic degeneration

There are rare reports of degenerative cyst formation at the tip of the herniated cerebellar tonsil in patients with Chiari 1 malformation. Koga [7] and Pueyrrdon [8] reported on the histological analyses of cystic cerebellar tonsils in Chiari 1 malformation, with results supporting the development of ischaemia likely through disturbance of blood supply. On close surveillance of a single patient across a 3-year period, where initial imaging showed no cerebellar tonsillar ischaemic or cystic changes, there was documented evolution of cerebellar tonsillar ischaemia at 8 months and eventual cerebellar cyst formation after a further 2 years, which further highlights the proposed stepwise pathogenesis.

The incidence of cystic cerebellar degeneration in cohorts of children with Chiari 1 malformation is reported at approximately 0.01% [4, 9]. There is however a reported high association (75%) of syringomyelia concurrent with and complicating cerebellar tonsillar cystic degeneration in children with Chiari 1 malformation [4].

Cerebellar tonsillar ischaemia and cysts can be observed prospectively on pre-operative MRI. Prospective identification of the changes however has previously been inconsistent, with as low as 33% (1/3) of cystic cerebellar lesions recognised on preoperative MRI [9].

### Holocord syringomyelia

Syringomyelia is a condition characterised by fluid-containing cavities within the spinal cord parenchyma, and the syringomyelia that accompanies posterior fossa abnormalities is considered a consequence of altered CSF flow dynamics [10]. The exact pathogenesis of these collections remains speculative. Although characteristically present within the cervicothoracic cord between C2 and T9, the lesion may be present throughout the cord and extend to the conus medullaris. These fluid collections may or may not communicate with the central spinal canal.

Reports of MRI prevalence of Chiari 1 malformation with syringomyelia in children are as low as 1.2% in girls and 0.5% in boys [11]. Through the literature, the incidence of syringomyelia in the entire Chiari 1 malformation patient cohort varies between 23 and 85% [12, 13], which probably infers the temporal relationship of herniation and compression to progressive syrinx development.

Patients with Chiari 1 malformation are subdivided according to the presence of a syrinx, with the treatment of symptomatic Chiari 1 malformation being neurosurgical as there is no effective non-surgical alternative to posterior fossa decompression. Incidental findings and asymptomatic patients would be kept under close surveillance. Patients were more likely to be symptomatic if syringomyelia was present, given its association with a greater degree of tonsillar descent and resultant disturbance of CSF flow dynamics [11], and unsurprisingly older children were more symptomatic.

### Neuropathic osteoarthropathy

An unusual pathology of painless destruction of weight-bearing bones and joints was caused by neurosensory deficit [14, 15] from pathologies involving the spinal cord or peripheral nerves—with the most common causes of neuropathic osteoarthropathy reported in the literature including diabetes, tabes dorsalis (syphilis), leprosy and syringomyelia [16–20]. In the paediatric population, alternative causes include congenital pain insensitivity and hereditary peripheral sensorimotor neuropathies [21].

Although syringomyelia is the main cause for neuropathic degeneration of the shoulder (6% of syringomyelia cases [22]), on occasion, it is the culprit for lower limb neuropathic osteoarthropathies including the calcaneus. The development of the expansive fluid-containing cavities within the spinal cord characteristic of syringomyelia leads to progressive spinal cord compression and abnormal nerve conduction [21]. In the case of Chiari I malformation, this is often exacerbated by spinal cord compression from the herniated cerebellar tonsils, especially in the instance of cystic degeneration such as our case report.

Involvement of the ankle and foot is commonly associated with spontaneous calcaneal fractures and is often atraumatic or with an otherwise innocuous antecedent trauma [17]. Stress injuries manifest from the sensory neuropathy and loss of protective sensation, with patients typically asymptomatic of load-related pain, and with continued overuse or superadded acute trauma, these stress injuries progress to fracture [18, 23]. There is often coincident autonomic neuropathy which causes increased blood flow and secondary bone resorption.

This process is radiologically manifest by both hypertrophic and atrophic patterns and is often admixed with either pattern present for both spinal and peripheral nerve causes [21]. Classic hypertrophic changes are characterised by osseous sclerosis and fragmentation, joint destruction and osteophyte formation. On the contrary, the atrophic form has an appearance of osseous resorption. Joint disorganisation with accompanying sanguineous joint and bursal effusions is typical of both forms.

### Summary

Type I Chiari malformations are a typically benign and asymptomatic entity that are mostly found incidentally with neuroimaging. This report describes a type I Chiari malformation complicated by cystic cerebellar tonsillar degeneration causing further dorsal cervical cord compression and holocord syrinx formation. This is highly likely to be the cause of the presenting neuropathic osteoarthropathy of the calcaneal body and apophysis.

Suboccipital decompressive craniectomy and upper cervical laminectomy are the optimal treatment for such lesions, with the addition of cystic cerebellar tonsillar resection as in this case report.
